# Takotsubo cardiomyopathy in a patient with esophageal cancer: a case report

**DOI:** 10.1186/1752-1947-2-379

**Published:** 2008-12-08

**Authors:** Tara C Gangadhar, Elisabeth Von der Lohe, Stephen G Sawada, Paul R Helft

**Affiliations:** 1 Department of Medicine, Indiana University Medical Center, Indianapolis, IN 46202, USA; 2 Indiana University School of Medicine Indiana University Medical Center, Indianapolis, IN 46202, USA; 3 Division of Cardiology, Indiana University Medical Center, Indianapolis, IN 46202, USA; 4 Division of Hematology/Oncology, Indiana University Medical Center, Indianapolis, IN 46202, USA

## Abstract

**Introduction:**

Takotsubo cardiomyopathy has increasingly been reported in the medical literature in recent years. Much is still unknown regarding risk factors and clinical relationships. We contribute this case report to the growing set of literature on the topic.

**Case presentation:**

We report the case of a 64-year-old woman with esophageal cancer who developed takotsubo cardiomyopathy, a form of reversible heart failure, and we present a review of the literature. Patients present with symptoms similar to an acute coronary syndrome; however, cardiac catheterization reveals patent coronary arteries, and symptoms of heart failure resolve completely within weeks.

**Conclusion:**

It is important that clinicians consider takotsubo cardiomyopathy in the differential diagnosis of heart failure and gain a basic understanding of the clinical presentation and diagnosis.

## Introduction

Takotsubo cardiomyopathy, also known as stress-induced cardiomyopathy, has increasingly been reported in the medical literature in recent years. While case series outlining the clinical features of the disease have now been published, much is still unknown regarding risk factors and clinical relationships. We contribute this case report to the growing set of literature on the topic.

## Case presentation

A 64-year-old woman with unresectable squamous cell carcinoma of the mid esophagus was treated with definitive chemoradiotherapy, including 4500 cGy of external beam radiotherapy and two cycles of cisplatin and a continuous venous infusion of 5-fluorouracil. Three months later, she was admitted to the hospital with new onset of choking with both solid and liquid foods. Her past medical history included generalized anxiety disorder and hypercholesterolemia. She had an extensive smoking history, but no known history of cardiac disease. A barium esophagram on admission revealed a tracheo-esophageal fistula for which she underwent a successful endoscopic stent placement with a self-expanding metallic stent.

**Figure 1:**
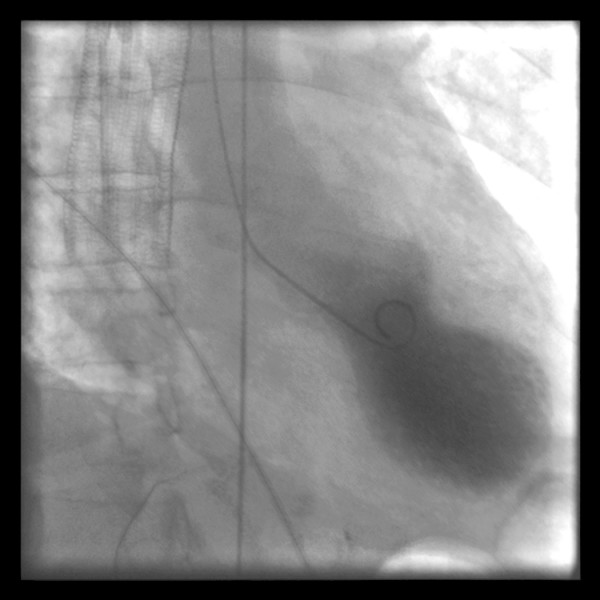
**Left ventriculography during systole showing apical ballooning akinesis with basal hyperkinesis in a characteristic takotsubo ventricle**.

**Figure 2:**
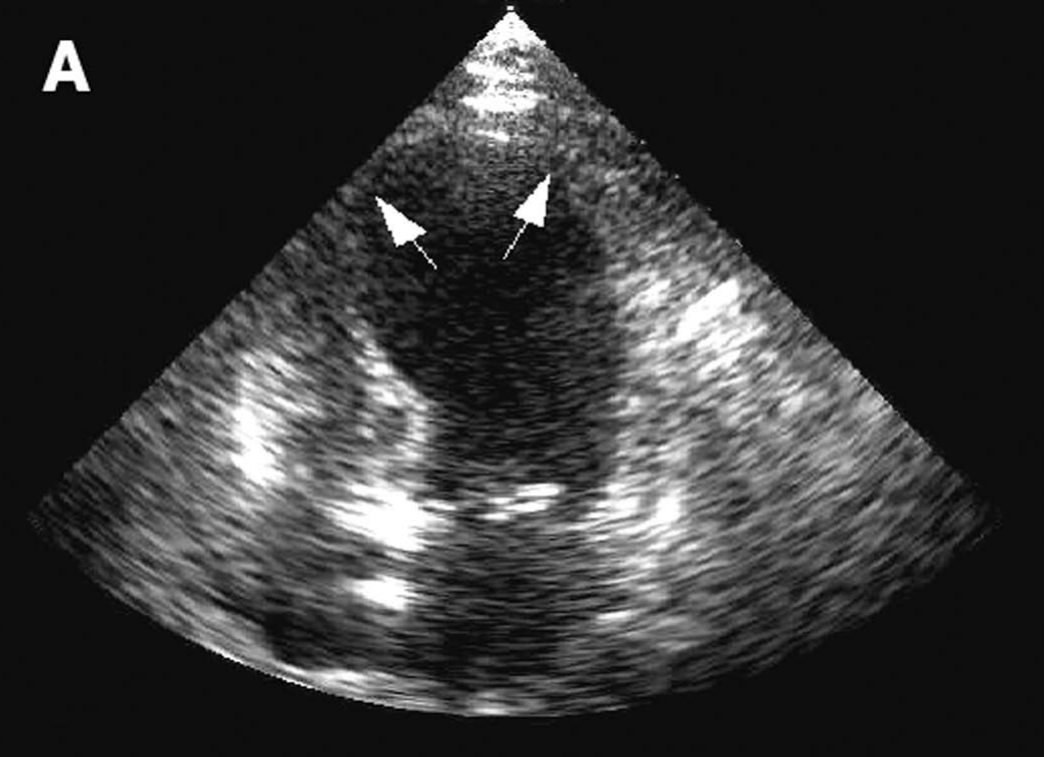
**Echocardiograph showing dilatation of the left ventricle in the acute phase**.

**Figure 3:**
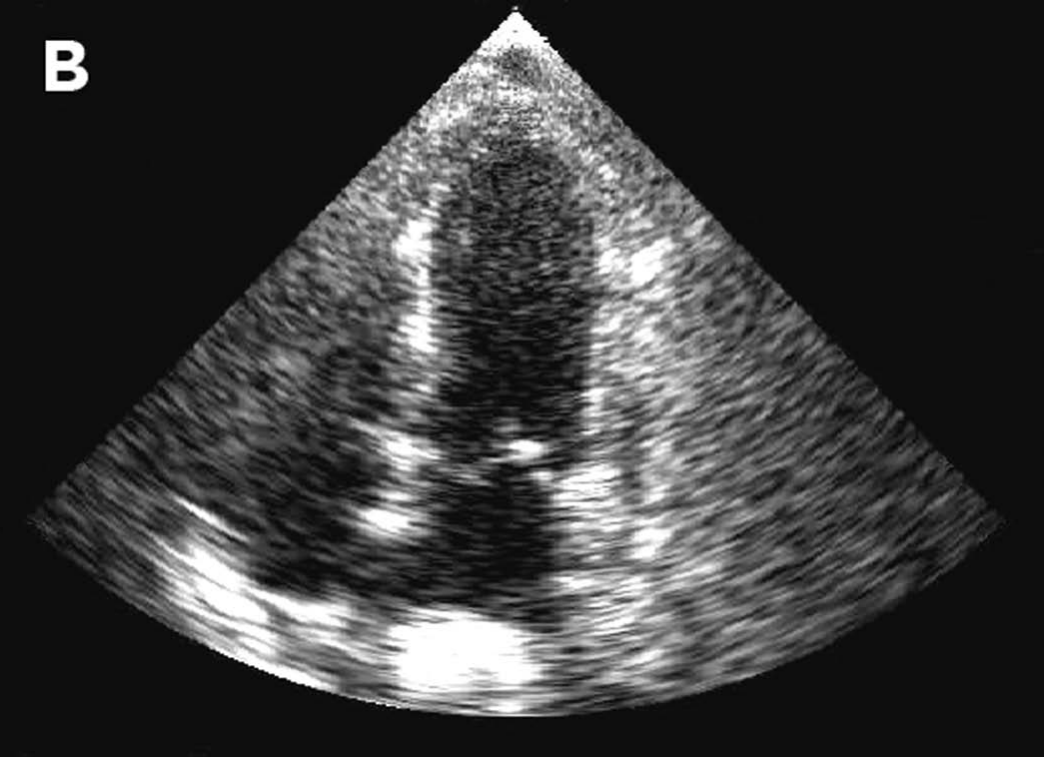
**Resolution of left ventricular function on repeat echocardiograph 6 days later**.

One day after the procedure, the patient developed substernal chest pain. Serial electrocardiograms revealed ST elevation in the anterior and lateral leads. Troponin measurements rose to 12.3 ng/ml; serum creatine kinase MB peaked at 10.6 ng/ml. Echocardiography revealed severely reduced global left ventricular systolic function and normal basal systolic function. Regional wall motion abnormalities were noted in the left anterior descending, left circumflex and right coronary artery distributions. The patient developed clinical signs of left-sided heart failure, including acute hypoxemic respiratory failure, and required intubation and mechanical ventilation. An emergency cardiac catheterization revealed normal, patent coronary arteries. A left ventriculogram revealed apical dilation of the left ventricle with akinesis of the whole ventricle except for the anterior and posterior base (Figure 1). Cardiology consultants felt that the patient's clinical and echocardiographic features met the diagnostic criteria for takotsubo cardiomyopathy [1]. The patient was managed conservatively with diuresis and had a rapid clinical improvement; she was extubated after 2 days. She was found to have marked improvement in regional wall motion and left ventricular systolic function on a repeat echocardiogram 6 days later (Figures 2 and 3). She had no clinical signs of congestive heart failure during follow-up 2.5 weeks later, making an ischemic or radiation induced irreversible cardiomyopathy unlikely.

## Discussion

Takotsubo cardiomyopathy is a rare form of reversible heart failure that has been reported primarily in Japan [2-4] and, more recently, in Europe and the United States [5,6]. Several cases have been described previously but, to the best of the authors' knowledge, this is the first full case report in a patient with cancer. The name of the syndrome derives from the classic appearance of the left ventricle, with an akinetic apex and hyperkinetic base, which takes the shape of a Japanese octopus fishing pot, or *takotsubo*. Also known as an apical ballooning syndrome, this cardiomyopathy occurs primarily in women in the seventh and eighth decades of life and is often associated with an acute physiological or emotional stress.

Patients present clinically with an acute coronary syndrome, frequently with chest pain at rest. ST elevations are present in electrocardiograms, most often in the precordial leads with evolutionary T wave inversions and corrected QT interval prolongation [1]. Serum laboratory studies reveal a moderate rise in cardiac enzymes. Unlike patients with acute coronary syndrome, coronary angiography usually reveals patent coronary arteries with no significant luminal stenosis or thrombosis. As in our patient, left ventriculography demonstrates an akinetic apex and hyperkinetic left ventricular base. According to proposed criteria for diagnosis [1], takotsubo cardiomyopathy is characterized by presentation and electrocardiographic changes similar to those caused by myocardial infarction; however, atherosclerotic coronary artery disease is usually absent and the transient left ventricular apical akinesis is usually beyond the distribution of a single coronary artery. An emotional or physiological stressor is identified in almost all cases. These findings are followed by a prompt resolution of symptoms with complete echocardiographic and clinical recovery of cardiac pump function within 2 to 3 weeks of presentation.

The etiology of takotsubo cardiomyopathy is not well understood, but is thought to be related to stress. Ueyama et al. have demonstrated reversible left ventricle apical ballooning in an animal model of emotional stress, rat immobilization [7,8]. In line with a stress-related mechanism, catecholamine-induced myocardial injury has been proposed, with elevated norepinephrine levels noted in many patients [9]. Others have proposed microcirculatory dysfunction as a possible mechanism of disease [10].

Treatment for takotsubo cardiomyopathy consists of supportive care of the patient during the period of reversible heart failure with hemodynamic support and appropriate treatment of complications, most commonly left ventricular dysfunction and arrhythmias. The prognosis of patients with takotsubo cardiomyopathy is good, with low in-hospital mortality and no reported increased risk of cardiac or other long-term morbidity or mortality [1,9].

## Conclusion

Although takotsubo cardiomyopathy has increasingly been reported in recent years, the prevalence is likely underestimated because of the low level of awareness and infrequent diagnosis. While suspicion of stress-induced cardiomyopathy does not justify withholding reperfusion therapy when indicated, increasing awareness among physicians will improve recognition and clinical management. Furthermore, adding to the literature provides a broader reference for studying clinical relationships in this illness and highlights its underestimated prevalence.

## Consent

Written informed consent was obtained from the patient's next-of-kin for publication of this case report and accompanying images. A copy of the written consent is available for review by the Editor-in-Chief of this journal.

## Competing interests

The authors declare that they have no competing interests.

## Authors' contributions

TG and PH conceived of the case report and drafted the manuscript. EV and SS performed and interpreted diagnostic studies, provided images for figures, and critically revised the manuscript for important intellectual content. All authors read and approved the final manuscript.
